# Falls at the Geriatric Hospital Ward in the Context of Risk Factors of Falling Detected in a Comprehensive Geriatric Assessment

**DOI:** 10.3390/ijerph191710789

**Published:** 2022-08-30

**Authors:** Lukasz Magnuszewski, Aleksandra Wojszel, Agnieszka Kasiukiewicz, Zyta Beata Wojszel

**Affiliations:** 1 Department of Geriatrics, Faculty of Health Sciences, Medical University of Bialystok, 15-471 Bialystok, Poland; 2 Department of Geriatrics, Hospital of the Ministry of Interior and Administration in Bialystok, 15-471 Bialystok, Poland; 3 Doctoral Studies, Faculty of Health Sciences, Medical University of Bialystok, 15-471 Bialystok, Poland; 4 Student’s Scientific Society at the Department of Geriatrics, Faculty of Health Sciences, Medical University of Bialystok, 15-471 Bialystok, Poland

**Keywords:** in-patient fall, fall risk factors, fall detection, geriatric patients, fall prevention

## Abstract

It is only by knowing the most common causes of falls in the hospital that appropriate and targeted fall prevention measures can be implemented. This study aimed to assess the frequency of falls in a hospital geriatrics ward and the circumstances in which they occurred and evaluate the parameters of the comprehensive geriatric assessment (CGA) correlating with falls. We considered medical, functional, and nutritional factors associated with falls and built multivariable logistic regression analysis models. A total of 416 (median age 82 (IQR 77–86) years, 77.4% women) hospitalizations in the geriatrics ward were analyzed within 8 months. We compared the results of a CGA (including health, psycho-physical abilities, nutritional status, risk of falls, frailty syndrome, etc.) in patients who fell and did not fall. Fourteen falls (3.3% of patients) were registered; the rate was 4.4 falls per 1000 patient days. They most often occurred in the patient’s room while changing position. Falls happened more frequently among people who were more disabled, had multimorbidity, were taking more medications (certain classes of drugs in particular), had Parkinson’s disease and diabetes, reported falls in the last year, and were diagnosed with orthostatic hypotension. Logistic regression determined the significant independent association between in-hospital falls and a history of falls in the previous 12 months, orthostatic hypotension, Parkinson’s disease, and taking statins, benzodiazepines, and insulin. Analysis of the registered falls that occurred in the hospital ward allowed for an analysis of the circumstances in which they occurred and helped to identify people at high risk of falling in a hospital, which can guide appropriate intervention and act as an indicator of good hospital care.

## 1. Introduction

Fall prevention in a hospital ward is one of the most critical components of in-patient care for older patients. One overarching goal of a skilled interdisciplinary team is to ensure patient safety and avoid adverse events [1]. Falls can affect a high percentage of community-dwelling 75+-year-old people, of which 45.1% report at least one fall in the past year [2]. A quarter of a century ago, 10 to about 25% of geriatric patients fell while in hospital [3,4]. Epidemiological data suggest that the incidence of falls is not so high among older hospital patients [5]. However, we should look at them from the perspective of the patient’s short stay and regard them as ‘never events’ for patients in a hospital.

Economic and social repercussions for frail patients and their families are severe [6]. An older person’s fall can result in disability, restriction of activity, and the fear of falling, which may reduce their quality of life and independence.

From a formal and legal point of view, one may perceive a hospital fall as a failure to provide the patient with the so-called safety of stay, which may determine the organizational guilt of the medical facility. Increasingly more often, patients in Poland claim compensation for damages resulting from such events [7]. We can assume that the situation will become even more urgent when institutions financing medical services cease to cover the costs of treating the consequences of falls in hospitals, as is the case in the United States [8]. Therefore, falls in hospitalized patients are an urgent safety issue in Poland.

Although falls occur in all wards of Polish hospitals [9], there are only a few publications on this topic. However, limited evidence demonstrates their correlates and the effectiveness of generally used preventive interventions, which mainly concern geriatric wards [5,10]. The risk of falls is assessed in these wards as part of a comprehensive geriatric evaluation and preventive measures are proposed for high-risk patients. Usually, incidents of falls are registered, and efforts are being made to minimize the risk of further accidents.

Falls are multifactorial and can result from intrinsic and extrinsic factors. The former include diseases, medications, and physical and mental disabilities. The latter include environmental factors, home hazards, or unfriendly hospital routines. Therefore, fall prevention is all about the management of internal risk factors (e.g., problems with walking and use of psychoactive drugs, confusion, blurred vision, dizziness, and frequent use of the toilet) and optimization of the ward routine, and the design of hospital environment [11,12].

Because the problem of falls in Polish hospitals seems to be underestimated and not thoroughly researched, our study aimed to identify the frequency of falls in a geriatric hospital ward. Furthermore, we attempted to assess the circumstances in which they occurred (time, place, activity, risk factors) and evaluate the comprehensive geriatric assessment parameters correlated with falls. The secondary aim was to propose changes in the fall prevention procedure in the ward based on these findings to make it more precise and effective.

## 2. Materials and Methods

We analyzed the medical records of all patients participating in a prospective cross-sectional study on the functional health status of geriatric in-patients [13]. Furthermore, we matched this data with the geriatrics ward patient register of falls documented for patients admitted to the ward between 1 September 2014 and 30 April 2015. The Department of Geriatrics of the Hospital of the Ministry of Interior in Bialystok, Poland, admits patients over 60, mainly in a planned manner, to provide them with a comprehensive geriatric assessment, reduction of polytherapy, and a long-term care plan.

The focus of this study was on in-hospital falls. Therefore, we adopted the World Health Organization’s definition of a fall: “inadvertently coming to rest on the ground, floor or other lower level, excluding intentional change in position to rest in furniture, wall or other objects” [14], which occurred during the patient’s stay in the hospital. So, we did not include slips, trips, or near misses in the analysis; these incidents are generally not reported by patients and nurses in the ward register of falls.

### 2.1. Prevention Strategies and Interventions Implemented at Geriatric Ward

At our department, we follow the national fall prevention standards, as detailed in the study by Mazur et al. [5]. Based on a comprehensive geriatric assessment, an individualized multifactorial approach to reducing patient fall risk was applied. In all patients admitted, the physiotherapist records the history of falls in the last 12 months and assesses the gait, balance, muscle strength, prevalence of orthostatic hypotension, and orthopedic equipment/walking aids needed. In addition, visual and hearing abilities are tested, the cognitive functions of older patients and their emotional health status are assessed, and possible environmental hazards in the patient’s home are discussed. Finally, each patient is educated about ward routines and how to move around the ward safely.

All falls in the ward are registered, and nurses on medical duty collect patients’ vital signs shortly after the fall (blood pressure, heart rate, blood glucose level). They note pre-fall behavior, time and place of the fall, injuries, taken drugs that could be a cause, and hospitalization day. In addition, each patient receives fall prevention recommendations for use in the hospital and subsequent recovery at home regarding risk factors that increase the unfortunate event, and anti-fall prevention regarding movement, medications, safe home space preparation, bathroom adaptation, footwear, and orthopedic equipment for their individual needs.

### 2.2. Data Collected

We collected data on patients’ sociodemographic characteristics (age, sex, place of living—urban/rural) and medical information. The latter included data on 15 chronic diseases (hypertension, ischemic heart disease, chronic cardiac failure, peripheral arterial disease, myocardial infarction, atrial fibrillation, history of transient ischemic attack (TIA) or stroke, diabetes or prediabetes, chronic renal disease, chronic obstructive pulmonary disease, dementia, parkinsonism, neoplasm, chronic osteoarthritis, and osteoporosis). We also gathered information on the length of hospital stay, number of chronic diseases (we defined multimorbidity as 5 or more diseases of the 15 listed above), falls and hospitalizations in the last 12 months, blood pressure at admittance, and selected laboratory values (glomerular filtration rate—GFR, counted using the CKD-EPI formula, serum creatinine level, total cholesterol, low-density lipoprotein—LDL and high-density lipoprotein—HDL cholesterol, thyroid-stimulating hormone—TSH, vitamin B12, calcium, sodium, hemoglobin, albumin), and assessed the thromboembolic risk with the CHA_2_DS_2_-VASc scale.

We checked for medications that could influence the risk of falls (antidepressants; neuroleptics; pro-cognitive medications; benzodiazepines; antihypertensive drugs, antiarrhythmics; lipid-lowering drugs; non-steroidal anti-inflammatory drugs). We collected data on the number of medications taken at admittance and defined polypharmacy as five or more drugs taken [15].

We gathered data on the physical and mental abilities of an older person assessed within a routine comprehensive geriatric assessment. It included the Barthel index score (the ability to carry out 10 basic activities of daily living; Cronbach alfa = 0.935; min—0, max—100 points) [16], the Duke Older American Resources and Services (OARS) I-ADL score (6 items of instrumental activities of daily living; Cronbach alfa = 0.916; min—0, max—12 points) [17], Norton Scale score (risk of pressure sores; min—5, max—20 points) [18], the cognitive status assessment results (the Abbreviated Mental Test Score-AMTS; min—0; max—10 points) [19], and the emotional state (the 15-item Geriatric Depression Scale, GDS; min—0, max—15 points) [20].

Data on the risk of recurrent fall assessment included the Performance Oriented Mobility Assessment (POMA) score (min—0, max—28 points) [21], Timed Up and Go test (TUG) results [22], and gait speed measurement during a 4.57 m walk at usual pace (a patient was classified as slow when gait speed ≤0.8 m/s). We collected data on dynapenia (or probable sarcopenia), derived from the handgrip strength of the dominant hand (mean of two measurements, assessed using a manual hydraulic dynamometer SAEHAN DHD-1 and diagnosed in men if grip strength was lower than 27 kg and in women if it was lower than 16 kg [23,24]), and on patient’s frailty (assessed with the 7-item Canadian Study of Health and Aging Clinical Frailty Scale (CFS); patients were classified as frail if they belonged to category 5–7, pre-frail if they were in category 4, and robust in category 1–3 [25]). Additionally, we derived information on nutritional status (risk of malnutrition with Mini Nutritional Assessment-Short Form-MNA-SF (min—0, max—14 points) [26], body mass index—BMI, waist-hip ratio—WHR, calf circumference—CC, mid-arm circumference—MAC, albumin level, and hemoglobin level).

### 2.3. Statistical Analysis

We used STATISTICA 13.3 software package (TIBCO Software, Palo Alto, CA, USA) and the IBM SPSS Version 18 Software suit (SPSS, Chicago, IL, USA) to analyze the collected data. First, we assessed the distribution of variables with the Shapiro–Wilk test. We presented data as the medium (M) and standard deviation for normally distributed variables and as the median (Md) and interquartile range (IQR) for non-normally distributed ones. We presented the number of cases and percentage for categorical variables. We compared proportions using χ^2^ tests or Fisher’s exact test as appropriate while the Mann–Whitney U test and Kruskal–Wallis test by ranks were used to compare the distribution of continuous variables. We regarded a two-tailed *p* value of less than 0.05 as significant in all analyses. We omitted missing values and calculated the statistics for the adequately reduced groups. We included fatal cases in the analysis, as they did not exceed 1% of cases.

We performed a multivariable logistic regression analysis to determine the possibility of the prediction of falls, including predictors with a *p*-value less than 0.1 and excluding those highly correlated with avoiding multicollinearity. In the first step, we aimed to evaluate the possibility of using some simple tests associated with fall risk assessment and easily identifiable in clinical practice (making the model possible to be implemented straightforwardly) to predict falls in the hospital. We used a backward stepwise regression (Model 1 with a block of four predictors). Next, we controlled the influence of other variables that were significantly correlated with in-patient falls (Model 2 with an additional block of 16 predictors meeting the criterion *p* < 0.1). In the final Model 3, we retained only variables that were statistically significant at *p*-value < 0.05 (enter method). We reported ORs with 95% CIs and *p* values for each model parameter. Finally, we evaluated the statistical significance of the model with the Hosmer–Lemeshow goodness-of-fit C-statistics (significant *p* value indicating an overall lack of fit).

## 3. Results

### 3.1. Prevalence and Circumstances of In-Hospital Falls and Their Consequences

During the study period, 14 (3.3%) of 416 hospitalized patients experienced a fall during their hospital stay. The calculated rate was 4.4 falls per 1000 patient days. Four (<1%) patients deceased within the duration of this study, and none of the deaths were due to a hospital fall.

**Figure 1:**
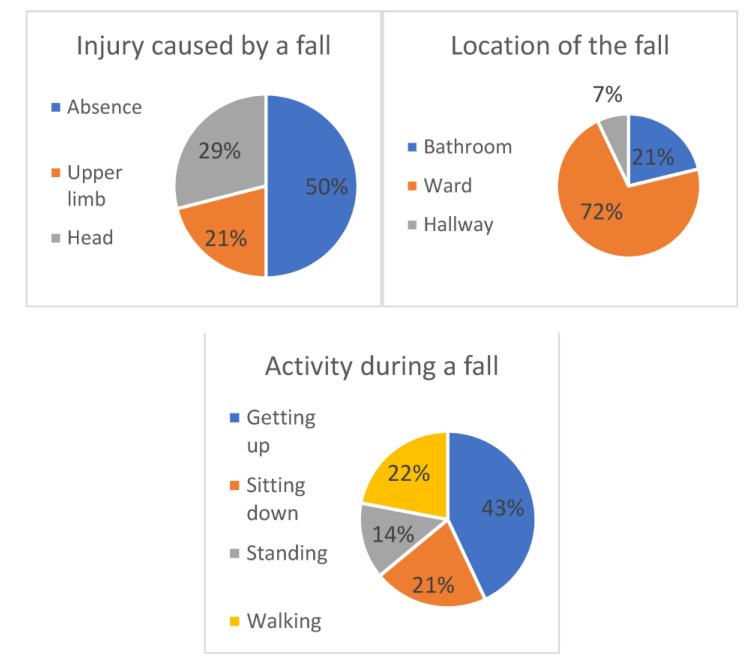
Circumstances of falls (location, activity, and injury).

**Figure 2:**
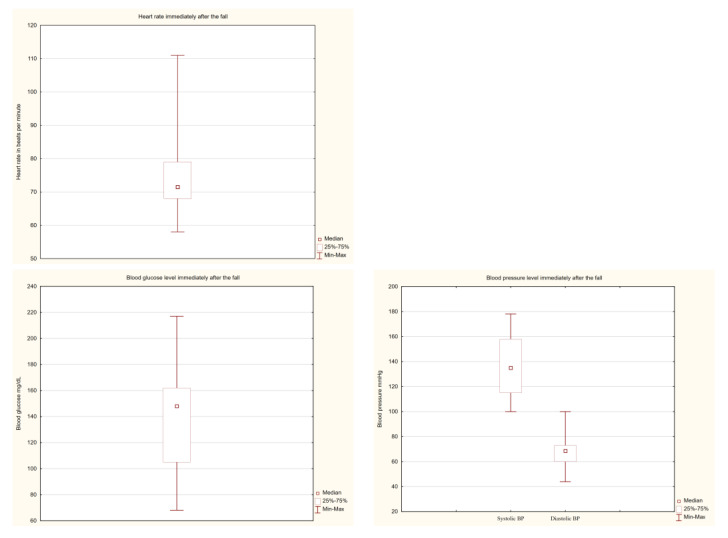
Basic parameters examined immediately after the fall.

Most falls occurred in patients’ rooms (Figure 1) and less often in the bathroom and hallway. The activity during which these incidents occurred most often was getting up from bed. Half of the accidents resulted in injury to the body (most often the head or upper limbs). The scatter of values for blood sugar, blood pressure, and heart rate, which were assessed just after the fall, was rather large (Figure 2).

### 3.2. Study Cohort Characteristics—Sociodemographic and Medical Correlates of Falls

The in-patient fallers’ and non-fallers’ sociodemographic and chosen medical characteristics are presented in Table 1. The study groups did not differ in age, sex, and place of living. Most of them were above 75 years (84.1%), female (77.4%), and living in towns (79.1%). In-patient fallers and non-fallers differed significantly in their length of stay in the ward (13.0 (IQR 8.8–17.0) days vs. 8.0 (IQR 5.0–9.0), *p* ≤ 0.001), percentage reporting falls in the previous year (90.9% of in-patient fallers vs. 42.4% of non-fallers, *p* = 0.01), number of chronic diseases (fallers: Md 6.5 (IQR 5.0–8.0) vs. non-fallers: Md 5.0 (IQR 3.0–6.0), *p* = 0.001), and prevalence of multimorbidity (92.9% vs. 56.2%, *p* = 0.004). Additionally, the prevalence of some medical problems was significantly higher in the fallers group: orthostatic hypotension (42.9% vs. 15.0% in non-fallers, *p* = 0.01), Parkinson’s disease (35.7% vs. 12.4% in non-fallers, *p* = 0.01), and diabetes (71.4% vs. 28.9% in non-fallers, *p* = 0.01). In the in-patient fallers group, the total cholesterol level was significantly lower (133.5 (112.0–165.0) mg/dL vs. 179 (147.0–213.0) mg/dL in non-fallers, *p* ≤ 0.001) in addition to LDL and HDL cholesterol levels, and the serum creatinine level was significantly higher. However, the percentage of chronic kidney disease and the prevalence of other chronic diseases were not substantially different in both groups. Patients who experienced a hospital fall had a significantly higher risk of thromboembolic complications as assessed using the CHA_2_DS_2_-VASc scale.

### 3.3. Study Cohort Characteristics—Pharmacotherapy Correlates of Falls

The in-patient fallers’ and non-fallers’ pharmacotherapy characteristics are presented in Table 2. Although the groups differed significantly in the median number of medications taken (fallers: 10.5 (6.0–12.0) vs. non-fallers: 7.0 (5.0–9.0), *p* = 0.01), the prevalence of polypharmacy was similar. The percentage of patients taking some medications was significantly higher in the in-hospital fallers group: insulin (35.7% vs. 6.6% in non-fallers, *p* < 0.001), metformin (50.0% vs. 14.8%, *p* < 0.001), statins (64.3% vs. 33.4%, *p* = 0.01), and benzodiazepines (28.6% vs. 10.4%, *p* = 0.03). This difference was on the verge of statistical significance in the case of neuroleptics (28.6% vs. 12.8% in non-fallers, *p* = 0.08).

### 3.4. Study Cohort Characteristics—Mental and Physical Abilities and Nutritional Correlates of Falls

Table 3 presents in-hospital fallers’ and non-fallers’ mental and physical abilities and nutritional parameters. The groups did not differ significantly in AMTS and GDS scores. The POMA results were significantly worse in the fallers’ group (Md = 17.0 (IQR 14.0–23.5) points vs. 23.0 (IQR 18.0–28.0) in non-fallers, *p* = 0.03). The handgrip strength did not differ significantly between in-hospital fallers and non-fallers. Additionally, we did not observe any difference between fallers and non-fallers in the percentage of patients with dynapenia. Most patients who experienced a fall in the hospital were significantly frailer according to the Clinical Frailty Scale; the level of frailty status classified with the CFS was significantly higher in fallers (Md = 5.5 (5.0–6.0) vs. 5.0 (4.0–5.0) in non-fallers, *p* = 0.01). The mean BMI and percentages of patients with BMI below 24.0 kg/m^2^ and BMI above 30 kg/m^2^ were not significantly different between the study groups. Additionally, the MNA-SF score did not differ significantly.

### 3.5. Study Cohort Characteristics—Abilities in Performing Activities of Daily Living Correlates of Falls

The Barthel index and the Duke OARS scale median scores were lower in the fallers group, although the differences were not statistically significant. Table 4 shows the differences in the individual basic activities of daily living (ADL) assessed with the Barthel index and advanced ADL assessed by the QARS I-ADL questionnaire. Patients who experienced a hospital fall significantly more often reported needing help in bathing, toilet use, doing housework, going shopping, and disposing of their money.

### 3.6. Prediction of Falls in a Multivariable Logistic Regression Analysis

We performed a multivariable logistic regression analysis on falls in the hospital as a dependent variable. In the first step, we included in the regression Model 1 a block of four predictors: history of falls in the last 12 months, a positive test for orthostatic hypotension, result in the Clinical Frailty Scale, and result in POMA test, as shown in Table 5. Three of these variables, but not POMA, was significant in a backward analysis. As a result, the overall success rate for the model was 96.7%, with very high specificity of 100% and very low (0%) sensitivity (Nagelkerk’s R^2^ = 0.208). In the next step, we added to this model a block of another 16 patient characteristics that were significantly correlated with in-patient falls in the univariable analyses: Barthel index, IADL, sex, diabetes mellitus, Parkinson’s disease, number of conditions, taking statins, insulin, sulfonylurea, metformin, quetiapine, neuroleptics, benzodiazepines, SSRI, serum level of cholesterol, and creatinine. The backward stepwise regression in Model 2 confirmed the significance of four of these variables: Parkinson’s disease, statin, benzodiazepine, and insulin use, and it was found that the CFS variable was statistically insignificant. The overall success rate for the model was 98.2%, with the same specificity (100%) as Model 1 and better (44.4%) sensitivity (Nagelkerk’s R^2^ = 0.579). The Hosmer–Lemeshow goodness-of-fit *p*-values for Model 1 and Model 2 were above 0.05, leading to rejection of the null hypothesis of a lack of fit.

Based on Models 1 and 2, we created the final multivariable logistic regression model of the determinants of in-hospital falls, using the enter method, which included only six relevant, statistically significant variables (presented in Table 6). The Hosmer–Lemeshow *p*-value confirmed it fitted well, and Nagelkerk’s R^2^ was 0.541, similar to that of Model 2.

## 4. Discussion

This study aimed to evaluate the prevalence of in-hospital falls, their circumstances, and risk factors in geriatric ward patients. Our results confirmed other authors’ observations that nowadays, falls occur only in a small percentage of people hospitalized in a geriatrics ward (3.3%), even though it is a group with a high frequency of characteristics that are considered important fall risk factors [27,28]. It may reflect the changes that take place in the care of elderly patients in hospitals and indicate the effectiveness of fall prevention principles implemented over the years in geriatric wards. Although, one should also consider the possible impact of differences in defining hospital falls. According to the literature, in-hospital fall rates are between 2.2 and 17.1 falls per 1000 patient days, with the highest rates in geriatric wards [29,30]. In our department of geriatrics, the rate was 4.4 falls per 1000 patient days. The comparison of in-patient fall rates adjusted for patient-related fall risk factors that are not modifiable by care can serve as an indicator of the quality of care provided by a hospital/ ward [31].

Falls in older adults are considered one of the geriatric syndromes because of their high prevalence, multiple causes, reoccurrence, and serious consequences. The majority of hospital falls observed in our study occurred while getting up (43%) or walking (22%) in a patient’s room (71%) or bathroom (21%). Half of them resulted in an injury requiring medical intervention, and the hospital length of stay of patients that experienced in-hospital falls was significantly longer. Struble-Fitzsimmons described the location and activity of patients related to falls in the department of geriatric psychiatry. Most of the 61 falls, 67.8%, occurred in the patient’s room (44.1%), the bathroom (23.7%), and the corridor (18.6%). The most common activities of the patients during the fall were walking (49.1%) and shifting (36.8%, n = 21). More than half (57.1%) of all transfer-related falls occurred while getting out of bed [32]. Draper et al., who researched 55 people who fell in hospital, found that the most common location of falls was the patient’s room, and the second most common location was the ward, followed by the bathroom and the corridor [33]. These results largely reflect the data from our department.

In our study, in-hospital falls were significantly more frequent among people dependent on others during basic and instrumental activities of daily living, with multimorbidity and taking more medications (certain classes of drugs in particular, such as statins, benzodiazepines, or antidiabetics) and reporting falls in the last year. They were more often diagnosed with Parkinson’s disease, diabetes, orthostatic hypotension, lower cholesterol (a result of taking statins more often but also a consequence of malnutrition), and higher serum creatinine. The characteristics of gait and balance measured by the POMA score were significantly worse in the in-patient fallers group. They were also frailer according to the CFS scale.

The fact that 1/4 of the falls occurred in the bathroom and 3/4 of those who experienced a fall were dependent on others for bladder control and using the toilet or bathroom demonstrates the importance of properly securing the use of the bathroom by elderly patients in the geriatric ward. It is worth mentioning that the housing conditions were not optimal. All patients shared toilets and showers, which could also have influenced these results. Most of the falls in our ward occurred while the patient was standing up, which could be directly related to orthostatic hypotension [34,35]. This is confirmed by the fact that orthostatic hypotension was an independent predictor of in-hospital falls in the logistic regression models.

No significant association with being a faller was observed in our study for many other recognized risk factors for falls. However, this may be due to the geriatric ward patients’ specificity (more advanced age, predominance of women, more advanced disability, and severity of medical problems), as we observed in our previous analyses [13]. So, for instance, various studies confirmed that more advanced age, female sex, dementia, stroke, or taking specific types of medications (e.g., dementia drugs or antidepressant medications) increased the risk of falls. Still, we did not observe this in our study. Whereas, for instance, a study by de Carle and coworkers found that female sex was a risk factor for falling in in-patient geriatric psychiatry [36] and Struble-Fitzsimmons’ analysis revealed that females were more than twice as likely to fall compared to males [32]. Our study did not observe any association between hospital falls and dementia or cognitive impairment in psychological tests. In a study by Zhang X-M and co-workers, in-patients with mental frailty were at greater risk of falls than non-frail and cognitively intact patients [37]. Several studies suggested that nutritional status and BMI should be evaluated when assessing the risk for hospital falls in older age. O’Neil and co-workers found an association between low BMI (≤18.5 kg/m^2^) and increased risk of hospital falls [38], and in the study by Mazur et al., lower BMI (<23.5 kg/m^2^) appeared to be an in-hospital fall risk factor [5]. Decreased BMI may be related to frailty, a syndrome prevalent in geriatric in-patients and associated with increased fall risk [37,39]. However, we did not observe this association in our study. Although, we observed falls more often in more frail patients. Still, we must remember that obesity is also related to sarcopenia and frailty [40].

Many researchers are still looking for the best tool for in-patient fall risk screening [41]. We also attempted to assess whether combining a few simple methods of estimating the risk of falling can increase their predictive ability. In our study, the POMA—but not TUG—test correlated significantly with in-hospital falls, but the regression analysis did not confirm POMA as an independent predictor of in-hospital falls. It is worth remembering that all these instruments have limitations on their predictive value or practicability [30,42,43]. POMA has limited use as it only assesses gait and balance, and the constraints associated with the severity of a patient’s health status may render the tool useless or workable only in patients with the lowest risk of falling. Large and co-workers showed that patients who were unable to perform the Timed Up and Go due to a non-physical disability had the highest fall rate, followed by those with a physical disability. In comparison, those who were able to perform the Timed Up and Go had the lowest fall rate. Acutely unwell and immobile patients with dementia and delirium were not at an excessive risk of falls [42]. Therefore, patients’ inability to complete the test can place them at a higher risk of falling. A good alternative to POMA or TUG could be the falls risk tool developed from interRAI AC Acute Care (AC) [44]. Attention is also drawn to the possibility of improving the predictive ability thanks to the dual-task versions [45]. However, it is also worth considering the appropriate choice of the tool [46].

As revealed by multivariable logistic regression, the most important independent predictors of falls in our group of geriatric patients were a positive history of falls within the previous 12 months, orthostatic hypotension diagnosed at the beginning of hospitalization, diagnosis of Parkinson’s disease, and taking benzodiazepines, statins, and insulin. However, the model built on these variables allowed for identification of people with no risk of falling in hospital rather than those who suffered a fall. Nevertheless, it points to patient characteristics that should be paid close attention during hospitalization. Indeed, an indispensable element of assessment of a geriatric patient should be the question about falling in the last 12 months, performing an orthostatic test, and paying attention to the medications taken [34]. Our study also confirmed the well-known fact that there is a very high risk in patients with Parkinsonian syndromes, who should be under special supervision. This is due not only to worse efficiency but also to an essential autonomic dysfunction, which is also manifested by transient orthostatic hypotension, which is of great importance when getting up from bed [47]. This may partly explain the results of our study.

The registration of falls conducted in the hospital ward allowed us to analyze the circumstances of their occurrence and potential risk factors that may guide the appropriate intervention. Diagnosis of the causes of falls and targeted interventions can reduce the risk and frequency of occurrence to prevent morbidity and mortality associated with falls. This is an element of the appropriate quality of care in a medical facility, which may also translate into economic results of its functioning.

The main limitation of this study is that it was a cross-sectional study based on retrospective data, and the study sample consisted of patients from only one geriatric ward, followed over a relatively short period of eight months. Hence, they cannot be generalized to the entire population of patients hospitalized in geriatric wards in Poland. Furthermore, the relatively small group of patients who fell in the hospital may also have affected the results. As our study was not a prospective clinical trial but a “usual practice” study, we included final patient clinical assessment results as part of the comprehensive geriatric assessment rather than scores for individual questions on different scales. Therefore, the data collected during this study did not allow evaluation of the Cronbach alpha or other psychometric properties of most scales (except for the Barthel index scores and Duke OARS IADL).

## 5. Conclusions

Our study confirmed that falls affect a relatively low percentage of people hospitalized in the geriatric ward. Still, half of the cases were associated with severe injuries and extended hospital stays, so they should be treated as ‘never events’ for patients in a hospital. Therefore, we need to pursue fall prevention much more aggressively. It is crucial to document falls and their circumstances in a given medical facility and analyze them from the perspective of risk factors typical of hospitalized patients. Our study showed that in the case of the geriatric ward, special attention and preventive measures should be directed at patients with a positive history of falls in the last year, diagnosed with orthostatic hypotension and Parkinson’s disease, and treated with statins, benzodiazepines, or insulin. It is worth extending these studies to other geriatric wards and assessing the results obtained in prospective analyses from the perspective of the effectiveness of preventive interventions.

## Figures and Tables

**Table 1:** Characteristics of study participants—sociodemographic and medical correlates of falls.

Characteristic	All	In-Patient Non-Fallers	In-Patient Fallers	*p* Value ^a^	Missing Values
No. (%) of patients	416 (100.0)	402 (96.7)	14 (3.3)	-	-
Age (y), Md (IQR)	82 (77.0–86.0)	82 (77.0–86.0)	81 (76.0–86.0)	0.78	-
Age (75+), *n* (%)	350 (84.1)	337 (83.8)	13 (92.9)	0.32	-
Sex (men), *n* (%)	94 (22.6)	88 (21.9)	6 (42.9)	0.07	-
Residence (rural), *n* (%)	87 (20.9)	85 (21.1)	2 (14.3)	0.41	-
Length of hospital stay, days, Md (IQR)	8 (5.0–9.0)	8 (5.0–9.0)	13 (8.8–17)	**<0.001**	-
Number of chronic diseases ^b^, Md (IQR)	5.0 (3.0–6.0)	5.0 (3.0–6.0)	6.5 (5.0–8.0)	**0.001**	-
Multimorbidity, *n* (%)	239 (57.5)	226 (56.2)	13 (92.9)	**0.004**	-
Falls in the last 12 months, *n* (%)	157 (43.9)	147 (42.4)	10 (90.9)	**0.001**	58
Hospitalization in the last 12 months, *n* (%)	122 (29.5)	116 (29.0)	6 (42.9)	0.20	3
Parkinson’s disease, *n* (%)	55 (13.2)	50 (12.4)	5 (35.7)	0.01	-
Dementia, *n* (%)	133 (32.0)	127 (31.6)	6 (42.9)	0.27	-
Depression, *n* (%)	181 (56.9)	172 (56.2)	9 (75.0)	0.12	98
Diabetes, *n* (%)	126 (30.3)	116 (28.9)	10 (71.4)	0.001	-
Hypertension, *n* (%)	327 (78.6)	315 (78.4)	12 (85.7)	0.39	-
Orthostatic hypotension, *n* (%)	57 (16.2)	51 (15.0)	6 (42.9)	**0.01**	63
Ischemic heart diseases, *n* (%)	223 (53.6)	213 (53.0)	10 (71.4)	0.13	-
Osteoporosis, *n* (%)	74 (17.8)	73 (18.2)	1 (7.1)	0.25	-
Osteoarthritis, *n* (%)	324 (77.9)	313 (77.9)	11 (78.6)	0.62	-
Chronic cardiac failure, *n* (%)	378 (90.9)	364 (90.6)	14 (100.0)	0.22	-
Atrial fibrillation, *n* (%)	98 (23.6)	95 (23.6)	3 (21.4)	0.57	-
Peripheral arterial disease, *n* (%)	64 (15.4)	61 (15.2)	3 (21.4)	0.36	-
Stroke/ TIA, *n* (%)	56 (13.5)	53 (13.18)	3 (21.4)	0.28	-
Rheumatoid arthritis, *n* (%)	20 (4.81)	20 (5.0)	0 (0.0)	0.39	-
Cholesterol- total, mg/dL, Md (IQR)	178 (146.0–212.0)	179 (147.0–213.0)	133.5 (112.0–165.0)	**<0.001**	27
LDL, mg/dL, Md (IQR)	118 (87.0–143.0)	120 (88.0–144.5)	76.0 (67.0–87.0)	**<0.001**	118
HDL, mg/dL, Md (IQR)	48 (39.0–57.0)	49 (40.0–58.0)	40.5 (32.0–42.0)	**<0.001**	101
Creatinin, mg/dL Md (IQR)	0.98 (0.84–1.19)	0.97 (0.84–1.18)	1.3 (0.9–1.41)	**0.04**	11
eGFR, mL/min/1.73 m^2^, M (SD)	57.9 (46.0–71.6)	58.1 (46.1–71.9)	49.4 (40.5–66.6)	0.13	11
TSH, mU/L, Md (IQR)	1.4 (0.82–2.1)	1.4 (0.82–2.1)	1.8 (1.2–2.3)	0.24	35
Vitamin B12, pg/mL, Md (IQR)	334.5 (242.6–430.6)	336.2 (242.8–430.6)	299.7 (221.8–343.8)	0.46	38
Na, mmol/L, Md (IQR)	140.0 (138.0–141.0)	140.0 (138.0–141.0)	142.0 (134.0–142.0)	0.60	10
Ca, mmol/L, Md (IQR)	4.6 (4.4–4.7)	4.6 (4.4–4.7)	4.5 (4.3–4.6)	0.17	102
Hemoglobin, g/dL, Md (IQR)	12.6 (11.5–13.7)	12.6 (11.5–13.7)	12.6 (11.3–13.2)	0.70	12
Albumin ≥ 35 mg/mL, *n* (%)	58 (14.9)	54 (14.4)	4 (28.6)	0.14	27
Systolic BP at admittance, mmHg, Md (IQR)	130.0 (120.0–140.0)	130.0 (120.0–140.0)	131.0 (120–140.0)	0.84	7
Diastolic BP at admittance, mmHg, Md (IQR)	70.0 (60.0–80.0)	70.0 (60.0–80.0)	70.0 (60.0–80.0)	0.41	7
CHA_2_DS_2_-VASc, Md (IQR)	4.0 (3.0–5.0)	4.0 (3.0–5.0)	5.5 (4.0–6.0)	**0.005**	2

^a^ x^2^ test or Fisher’s exact test, as appropriate, for categorical variables; Mann–Whitney test for continuous or interval variables. In all analyses a two-tailed *p*-value of less than 0.05 was regarded as significant (significant differences are in bold). ^b^ of 15 chronic diseases (hypertension, ischemic heart disease, chronic cardiac failure, peripheral arterial disease, myocardial infarction, atrial fibrillation, history of transient ischemic attack (TIA) or stroke, diabetes or prediabetes, chronic renal disease, chronic obstructive pulmonary disease, dementia, parkinsonism, neoplasm, chronic osteoarthritis, and osteoporosis); BP—blood pressure; Ca—serum calcium; eGFR—glomerular filtration rate; HDL—high-density lipoprotein; IQR—interquartile range; LDL—low-density lipoprotein; Md—median value; *n*—number of cases; Na—serum sodium; TIA—transient ischemic attack; TSH—thyroid-stimulating hormone.

**Table 2:** Characteristics of study participants—pharmacotherapy correlates of falls.

Parameter	All	In-Patient Non-Fallers	In-Patient Fallers	*p* Value ^a^	Missing Values
No. (%) of patients	416 (100.0)	402 (96.7)	14 (3.3)	-	-
Number of drugs at admittance, Md (IQR)	6.0 (5.0–9.0)	7.0 (5.0–9.0)	10.5 (6.0–12.0)	**0.01**	9
Polypharmacy, *n* (%)	322 (79.1)	309 (78.6)	13 (92.9)	0.17	9
α1-blockers, *n* (%)	25 (6.1)	23 (5.9)	2 (14.3)	0.19	10
ß-blockers, *n* (%)	258 (63.6)	250 (63.8)	8 (57.1)	0.40	10
Calcium channel blockers, *n* (%)	144 (28.1)	110 (28.1)	4 (28.6)	0.58	10
ACEI/ ARB, *n* (%)	222 (54.3)	212 (53.7)	10 (71.4)	0.18	7
Thiazide/ loop diuretics, *n* (%)	196 (42.3)	188 (48.0)	8 (57.1)	0.34	10
Aldosterone- receptor antagonists (spironolactone, eplerenone), *n* (%)	71 (17.5)	66 (16.8)	5 (35.7)	0.07	10
Statins, *n* (%)	142 (35.0)	133 (33.4)	9 (64.3)	**0.01**	10
Digoxin, *n* (%)	30 (7.4)	28 (7.1)	2 (14.3)	0.27	10
Antiarrhythmics, *n* (%)	9 (2.2)	9 (2.3)	0 (0.0)	0.56	10
Insulin, *n* (%)	31 (7.6)	26 (6.6)	5 (35.7)	**<0.001**	10
Metformin, *n* (%)	65 (16.0)	58 (14.8)	7 (50.0)	**<0.001**	10
Oral anti-diabetic drugs (SM, metformin), *n* (%)	90 (22.2)	82 (20.1)	8 (57.1)	**0.001**	10
Anti-diabetic drugs (SM, metformin, insulin), *n* (%)	105 (25.9)	96 (24.5)	9 (64.3)	**<0.001**	10
Thyroid hormones, *n* (%)	30 (7.4)	28 (7.1)	2 (14.3)	0.28	10
Cholecalciferol, *n* (%)	88 (21.7)	86 (22.0)	2 (14.3)	0.49	10
BDA, *n* (%)	45 (11.1)	41 (10.4)	4 (28.6)	0.03	9
SSRI, *n* (%)	115 (28.3)	108 (27.5)	7 (50.0)	0.07	10
Antidepressants (SSRI, mianserin), *n* (%)	133 (32.8)	126 (32.1)	7 (50.0)	0.13	10
Quetiapine, *n* (%)	49 (12.0)	45 (11.5)	4 (28.6)	0.05	10
Neuroleptics (quetiapine, haloperidol), *n* (%)	54 (13.3)	50 (12.8)	4 (28.6)	0.08	10
AChE-I (donepezil, rivastigmine), *n* (%)	41 (10.10)	38 (9.7)	3 (21.4)	0.15	10
Procognitive medications (AChEIs, memantine), *n* (%)	46 (11.3)	43 (11.0)	3 (21.4)	0.20	10

^a^ x^2^ test or Fisher’s exact test, as appropriate, for categorical variables; Mann–Whitney test for continuous or interval variables. In all analyses, a two-tailed *p*-value of less than 0.05 was regarded as significant (significant differences are in bold). ACE-Is—angiotensin-converting enzyme inhibitors; AChE-I—acetylcholinesterase inhibitor; ARB—angiotensin receptor blocker; BDA—benzodiazepine; IQR—interquartile range; Md—median value; *n*—number of cases; SM—sulphonylureas; SSRI—selective serotonin reuptake inhibitor.

**Table 3:** Characteristics of study participants—mental and physical abilities and nutritional correlates of falls.

Characteristic	All	In-Patient Non-Fallers	In-Patient Fallers	*p* Value ^a^	Missing Values
No. (%) of patients	416 (100.0)	402 (96.7)	14 (3.3)	-	-
AMTS, Md (IQR)	8.0 (6.0–9.0)	8.0 (6.0–9.0)	7.0 (6.0–8.0)	0.32	35
MMSE, Md (IQR)	21.0 (16.0–24.0)	21.0 (16.0–25.0)	19.0 (16.5–21.5)	0.40	231
GDS, Md (IQR)	7.0 (3.0–10.0)	6.5 (4.0–10.0)	8.5 (3.0–11.0)	0.36	52
POMA, Md (IQR)	23.0 (17.0–28.0)	23.0 (18.0–28.0)	17.0 (14.0–23.5)	**0.03**	94
POMA < 26, *n* (%)	199 (61.8)	188 (60.7)	11 (91.7)	**0.02**	94
POMA < 19, *n* (I%)	95 (29.5)	88 (28.4)	7 (58.3)	**0.02**	94
TUG, s, Md (IQR)	17.4 (11.9–28.0)	17.2 (11.7–27.7)	25.3 (20.7–29.1)	0.11	115
Gait speed, m/s, Md (IQR)	0.65 (0.4–0.96)	0.66 (0.4–0.59)	0.43 (0.32–0.63)	0.10	102
Gait speed ≤ 0.8 m/s, *n* (%)	166 (52.9)	158 (52.0)	8.0 (80.0)	0.08	102
Handgrip strength, kg, Md (IQR)	18.2 (13.7–22.8)	18.1 (13.8–22.8)	19.4 (13.2–22.5)	0.92	66
Dynapenia, *n* (%)	233.0 (66.6)	223.0 (66.2)	10.0 (76.9)	0.32	66
Norton scale, Md (IQR)	17.0 (15.0–19.0)	17.0 (15.0–19.0)	16.0 (15.0–18.0)	0.25	6
Clinical Frailty Scale, Md (IQR)	5.0 (4.0–5.0)	5.0 (4.0–5.0)	5.5 (5.0–6.0)	**0.01**	-
Clinical Frailty Scale classification				0.06	-
Robust, *n* (%)	62 (14.9)	62 (15.4)	0 (0)		
Pre-frail, *n* (%)	124 (29.8)	122 (30.3)	2 (14.3)		
Frail, *n* (%)	230 (55.3)	218 (54.2)	12 (85.7)		
Severe frailty (CFS = 6 or 7), *n* (%)	102 (24.5)	95 (23.6)	7 (50.0)	**0.02**	-
MNA-SF, Md (IQR)	12.0 (9.0–13.0)	12.0 (9.0–13.0)	12.0 (7.0–13.0)	0.54	12
MNA-SF score < 8, *n* (%)	198 (49.0)	192 (49.2)	6 (42.9)	0.42	12
BMI, kg/m^2^, M (SD)	28.5 (25.0–33.6)	28.5 (24.9–33.7)	30.3 (27.0–31.6)	0.55	62
BMI < 24 kg/m^2^, *n* (%)	66 (18.6)	65 (19.0)	1 (8.3)	0.31	62
BMI > 30 kg/m^2^, *n* (%)	148 (41.8)	141 (41.2)	7 (58.3)	0.18	62
WHR, Md (IQR)	0.90 (0.87–0.95)	0.90 (0.87–0.96)	0.93 (0.87–0.96)	0.45	63
MAC, cm, Md (IQR)	28.0 (26.0–30.0)	28.0 (26.0–30.0)	28.5 (26.0–30.0)	0.70	49
MAC ≤ 22 cm, *n* (%)	89 (24.3)	87 (24.7)	2 (14.3)	0.29	49
CC, cm, Md (IQR)	34.0 (31.5–37.0)	34.0 (31.0–37.0)	35.5 (33.0–40.0)	0.12	51
CC < 31 cm, *n* (%)	74 (20.3)	72 (20.5)	2 (14.3)	0.43	51

^a^ x^2^ test or Fisher’s exact test, as appropriate, for categorical variables; Mann–Whitney test for continuous or interval variables. In all analyses, a two-tailed *p*-value of less than 0.05 was regarded as significant (significant differences are in bold). AMTS—Abbreviated Mental Test Score; BMI—body mass index; CC—calf circumference; CFS—7-point Clinical Frailty Scale; IQR—interquartile range; Md—median value; MAC—mid arm circumference; MMSE—Mini Mental State Examination; MNA-SF—Mini Nutritional Assessment Short Form; *n*—number of cases; POMA—Performance Oriented Mobility Assessment; TUG—Timed Up-and-Go test.

**Table 4:** Characteristics of study participants—ADL and IADL correlates of falls.

Characteristic	All	In-Patient Non-Fallers	In-Patient Fallers	*p* Value ^a^	Missing Values
Barthel Index, Md (IQR)	90.0 (70.0–100.0)	90.0 (70.0–100.0)	77.5 (55.0–85.0)	0.06	6
Independent in:					
Bowel control, *n* (%)	360 (87.4)	346 (86.9)	14 (100.0)	0.15	4
Feeding, *n* (%)	343 (83.0)	332 (83.2)	11 (78.6)	0.65	3
Grooming, *n* (%)	333 (80.6)	332 (80.7)	11 (78.6)	0.84	3
Transferring (bed to chair and back), *n* (%)	297 (72.1)	289 (72.6)	8 (57.1)	0.20	4
Mobility on level surfaces, *n* (%)	292 (70.4)	284 (70.8)	8 (57.1)	0.27	1
Toilet use, *n* (%)	280 (67.8)	276 (69.2)	4 (28.6)	**<0.001**	3
Dressing, *n* (%)	276 (66.8)	270 (67.7)	6 (42.9)	0.05	3
Stairs managing, *n* (%)	227 (55.2)	219 (55.2)	8 (57.1)	0.88	5
Bladder control, *n* (%)	218 (53.0)	214 (53.9)	4 (28.6)	0.05	5
Bathing, *n* (%)	209 (21.4)	206 (51.9)	3 (21.4)	**0.02**	5
IADL, Md (IQR)	7.0 (3.0–11.0)	7.0 (3.0–11.0)	4.5 (2.0–8.0)	0.07	10
Independent in:					
Phone talk, *n* (%)	273 (66.8)	267 (67.6)	6 (42.9)	0.05	7
Taking medicines, *n* (%)	235 (57.5)	229 (58.0)	6 (42.9)	0.26	7
Disposing of money, *n* (%)	230 (56.4)	226 (57.4)	4 (28.6)	**0.03**	9
Making meals, *n* (%)	177 (43.7)	174 (44.5)	3 (21.4)	0.07	11
Going shopping, *n* (%)	126 (31.1)	125 (32.0)	1 (7.1)	**0.03**	11
Doing housework, *n* (%)	101 (25.0)	101 (25.8)	0 (0.0)	**0.02**	11

^a^ x^2^ test or Fisher’s exact test, as appropriate, for categorical variables; Mann–Whitney test for continuous or interval variables. In all analyses, a two-tailed *p*-value of less than 0.05 was regarded as significant (significant differences are in bold). ADL—activities of daily living; IADL—instrumental activities of daily living (DUKE OARS—Older Americans Resources and Services Program); IQR—interquartile range; Md—median value; *n*—number of cases.

**Table 5:** Predictors of falls—backward multivariable logistic regression models.

		Model 1			Model 2	
	OR	95% CI	*p* Value	OR	95% CI	*p* Value
Falls in the last 12 months	10.89	1.28–92.56	0.03	50.92	1.84–1412.6	0.02
Orthostatic hypotension	4.76	1.13–19.97	0.03	14.53	1.12–189.27	0.04
CFS	2.63	1.08–6.42	0.03	2.84	0.96–8.47	0.06
Parkinson’s disease				37.64	2.71–522.02	0.007
Statins				20.91	1.23–355.31	0.04
Insulin				56.79	3.66–880.59	0.004
Benzodiazepines				31.11	2.41–540.72	0.009
Negelkerk’s R^2^		0.208			0.579	
% correctly predicted		96.7%			98.2%	
sensitivity		0%			44.4%	
specificity		100%			100%	
Hosmer–Lemeshow goodness of fit		0.877			0.939	
		**Block 1**			**Block 2**	
Variables in block 1 and block 2	Falls in the last 12 months, orthostatic hypotension, POMA, CFS			Barthel index, IADL, sex, DM, Parkinson’s disease, number of diseases, statins, insulin, SM, metformin, quetiapine, neuroleptics, BDA, SSRI, cholesterol, creatinine		

BDA—benzodiazepines; CFS—Clinical Frailty Scale; CI—confidence interval; DM—diabetes mellitus; IADL—instrumental activities of daily living; OR—odds ratio; POMA—Performance Oriented Mobility Assessment; SM—sulfonylureas; SSRI—selective serotonin reuptake inhibitor.

**Table 6:** Predictors of falls—the final multivariable logistic regression model (enter method).

		Model 3	
	OR	95% CI	*p* Value
Falls in the last 12 months	41.74	2.47–705.95	0.010
Orthostatic hypotension	21.53	2.25–206.28	0.008
Parkinson’s disease	40.89	3.28–510.16	0.004
Statins	43.96	2.97–651.72	0.006
Insulin	34.76	3.33–362.75	0.003
Benzodiazepines	34.93	2.82–432.16	0.006
Negelkerk’s R^2^		0.541	
% correctly predicted		97.5%	
sensitivity		27.3%	
specificity		100%	
Hosmer–Lemeshow goodness of fit		0.703	

CI—confidence interval; OR—odds ratio.

## Data Availability

The data supporting the results in the current study are available from the corresponding author on reasonable request.

## References

[1] Zhang X., Huang P., Dou Q., Wang C., Zhang W., Yang Y., Wang J., Xie X., Zhou J., Zeng Y. (2020). Falls among older adults with sarcopenia dwelling in nursing home or community: A meta-analysis. Clin. Nutr..

[2] Wojszel Z.B., Bien B. (2004). Falls amongst older people living in the community. Rocz. Akad. Med. Bialymst..

[3] Nyberg L., Gustafson Y., Janson A., Sandman P.-O., Eriksson S. (1997). Incidence of falls in three different types of geriatric care. A Swedish prospective study. Scand. J. Soc. Med..

[4] Heinze C., Lahmann N., Dassen T. (2002). Frequency of falls in german hospitals. Gesundheitswesen.

[5] Mazur K., Wilczynski K., Szewieczek J. (2016). Geriatric falls in the context of a hospital fall prevention program: Delirium, low body mass index, and other risk factors. Clin. Interv. Aging.

[6] Burton E., Lewin G., O’Connell H., Hill K.D. (2018). Falls prevention in community care: 10 years on. Clin. Interv. Aging.

[7] Galeska-Sliwka A., Sliwka M. (2017). Liability for damages resulting from hospital falls. Wiad. Lek..

[8] Inouye S.K., Brown C.J., Tinetti M.E. (2009). Medicare nonpayment, hospital falls, and unintended consequences. N. Engl. J. Med..

[9] Mikos M., Banas T., Czerw A., Banas B., Strzępek Ł., Curyło M. (2021). Hospital Inpatient Falls across Clinical Departments. Int. J. Environ. Res. Public Health.

[10] Magnuszewski L., Swietek M., Kasiukiewicz A., Kuprjanowicz B., Baczek J., Wojszel Z.B. (2020). Health, Functional and Nutritional Determinants of Falls Experienced in the Previous Year-A Cross-Sectional Study in a Geriatric Ward. Int. J. Environ. Res. Public Health.

[11] van Loon I.N., Joosten H., Iyasere O., Johansson L., Hamaker M.E., Brown E.A. (2019). The prevalence and impact of falls in elderly dialysis patients: Frail elderly Patient Outcomes on Dialysis (FEPOD) study. Arch. Gerontol. Geriatr..

[12] de Sousa Costa A.G., de Araujo T.L., Cavalcante T.F., Lopes M.V.O., de Souza Oliveira-Kumakura A.R., Costa F.B.C. (2017). Clinical validation of the nursing outcome falls prevention behavior in people with stroke. Appl. Nurs. Res..

[13] Wojszel Z.B., Magnuszewski L. (2020). Type 2 Diabetes Correlates with Comorbidity and Nutritional Status but Not with Functional Health in Geriatric Ward Patients: A Cross-Sectional Study in Poland. Diabetes Metab. Syndr. Obes..

[14] Kalache A.F., Fu D., Yoshida S., Al-Faisal W., Beattie L., Chodzko-Zajko W., Fu H., James K., Kalula S., Krishnaswamy B. (2007). World Health Organisation Global Report on Falls Prevention in Older Age.

[15] Levy H.B. (2017). Polypharmacy Reduction Strategies: Tips on Incorporating American Geriatrics Society Beers and Screening Tool of Older People‘s Prescriptions Criteria. Clin. Geriatr. Med..

[16] Mahoney F.I. (1965). Functional evaluation: The Barthel index. Md. State Med. J..

[17] Fillenbaum G.G., Smyer M.A. (1981). The development, validity, and reliability of the OARS multidimensional functional assessment questionnaire. J. Gerontol..

[18] Norton D., McLaren R., Exton-Smith A.N. (1975). An Investigation of Geriatric Nursing Problems in Hospital.

[19] Hodkinson H.M. (1972). Evaluation of a mental test score for assessment of mental impairment in the elderly. Age Ageing.

[20] Yesavage J.A. (1988). Geriatric Depression Scale. Psychopharmacol. Bull..

[21] Tinetti M.E. (1986). Performance-oriented assessment of mobility problems in elderly patients. J. Am. Geriatr. Soc..

[22] Podsiadlo D., Richardson S. (1991). The timed “Up & Go“: A test of basic functional mobility for frail elderly persons. J. Am. Geriatr. Soc..

[23] Roberts H.C., Denison H.J., Martin H.J., Patel H.P., Syddall H., Cooper C., Sayer A.A. (2011). A review of the measurement of grip strength in clinical and epidemiological studies: Towards a standardised approach. Age Ageing.

[24] Cruz-Jentoft A.J., Bahat G., Bauer J., Boirie Y., Bruyère O., Cederholm T., Cooper C., Landi F., Rolland Y., Sayer A.A. (2019). Sarcopenia: Revised European consensus on definition and diagnosis. Age Ageing.

[25] Cheung A., Haas B., Ringer T.J., McFarlan A., Wong C.L. (2017). Canadian Study of Health and Aging Clinical Frailty Scale: Does It Predict Adverse Outcomes among Geriatric Trauma Patients?. J. Am. Coll. Surg..

[26] Kaiser M.J., Bauer J.M., Ramsch C., Uter W., Guigoz Y., Cederholm T., Thomas D.R., Anthony P., Charlton K.E., Maggio M. (2009). Validation of the Mini Nutritional Assessment short-form (MNA-SF): A practical tool for identification of nutritional status. J. Nutr. Health Aging.

[27] White A.M., Tooth L.R., Peeters G. (2018). Fall Risk Factors in Mid-Age Women: The Australian Longitudinal Study on Women‘s Health. Am. J. Prev. Med..

[28] Canning C.G., Paul S.S., Nieuwboer A. (2014). Prevention of falls in Parkinson‘s disease: A review of fall risk factors and the role of physical interventions. Neurodegener. Dis. Manag..

[29] Schwendimann R., Bühler H., De Geest S., Milisen K. (2006). Falls and consequent injuries in hospitalized patients: Effects of an interdisciplinary falls prevention program. BMC Health Serv. Res..

[30] Milisen K., Staelens N., Schwendimann R., de Paepe L., Verhaeghe J., Braes T., Boonen S., Pelemans W., Kressig R.W., Dejaeger E. (2007). Fall prediction in inpatients by bedside nurses using the St. Thomas‘s Risk Assessment Tool in Falling Elderly Inpatients (STRATIFY) instrument: A multicenter study. J. Am. Geriatr. Soc..

[31] Bernet N.S., Everink I.H., Schols J.M., Halfens R.J., Richter D., Hahn S. (2022). Hospital performance comparison of inpatient fall rates; the impact of risk adjusting for patient-related factors: A multicentre cross-sectional survey. BMC Health Serv. Res..

[32] Struble-Fitzsimmons D., Oswald A., DiPersia E. (2019). Patient Location and Mobility Factors Associated with Falls on an Inpatient Geriatric Psychiatry Unit. Act. Adapt. Aging.

[33] Draper B., Busetto G., Cullen B. (2004). Risk factors for and prediction of falls in an acute aged care psychiatry unit. Australas. J. Ageing.

[34] Wojszel Z.B., Kasiukiewicz A., Magnuszewski L. (2019). Health and Functional Determinants of Orthostatic Hypotension in Geriatric Ward Patients: A Retrospective Cross Sectional Cohort Study. J. Nutr. Health Aging.

[35] Menant J.C., Wong A.K.W., Trollor J.N., Close J.C.T., Lord S.R. (2016). Depressive Symptoms and Orthostatic Hypotension Are Risk Factors for Unexplained Falls in Community-Living Older People. J. Am. Geriatr. Soc..

[36] de Carle A.J., Kohn R. (2001). Risk factors for falling in a psychogeriatric unit. Int. J. Geriatr. Psychiatry.

[37] Zhang X.-M., Yuan L., Quo N., Bo H.-X., Jiao J., Wu X., Xu T. (2021). Cognitive Frailty and Falls in a National Cohort of Older Chinese Inpatients. J. Nutr. Health Aging.

[38] O’Neil C.A., Krauss M.J., Bettale J., Kessels A., Costantinou E., Dunagan W.C., Fraser V.J. (2018). Medications and Patient Characteristics Associated with Falling in the Hospital. J. Patient Saf..

[39] Schoene D., Kiesswetter E., Sieber C.C., Freiberger E. (2019). Musculoskeletal factors, sarcopenia and falls in old age. Z Gerontol. Geriatr..

[40] Yuan L., Chang M., Wang J. (2021). Abdominal obesity, body mass index and the risk of frailty in community-dwelling older adults: A systematic review and meta-analysis. Age Ageing.

[41] Scott V., Votova K., Scanlan A., Close J. (2007). Multifactorial and functional mobility assessment tools for fall risk among older adults in community, home-support, long-term and acute care settings. Age Ageing.

[42] Large J., Gan N., Basic D., Jennings N. (2006). Using the timed up and go test to stratify elderly inpatients at risk of falls. Clin. Rehabil..

[43] Köpke S., Meyer G. (2006). The Tinetti test: Babylon in geriatric assessment. Z Gerontol. Geriatr..

[44] Peel N.M., Jones L.V., Berg K., Gray L.C. (2021). Validation of a Falls Risk Screening Tool Derived from InterRAI Acute Care Assessment. J. Patient Saf..

[45] Vance R.C., Healy D.G., Galvin R., French H.P. (2015). Dual tasking with the timed “up & go” test improves detection of risk of falls in people with Parkinson disease. Phys. Ther..

[46] Kasiukiewicz A., Magnuszewski L., Swietek M., Wojszel Z.B. (2021). The Performance of Dual-Task Tests Can Be a Combined Neuro-Psychological and Motor Marker of Mild Cognitive Impairment, Depression and Dementia in Geriatric Patients—A Cross-Sectional Study. J. Clin. Med..

[47] Fanciulli A., Campese N., Goebel G., Ndayisaba J.P., Eschlboeck S., Kaindlstorfer C., Raccagni C., Granata R., Bonuccelli U., Ceravolo R. (2020). Association of transient orthostatic hypotension with falls and syncope in patients with Parkinson disease. Neurology.

